# Apolipoprotein E Deficiency Aggravates Neuronal Injury by Enhancing Neuroinflammation via the JNK/c-Jun Pathway in the Early Phase of Experimental Subarachnoid Hemorrhage in Mice

**DOI:** 10.1155/2019/3832648

**Published:** 2019-12-26

**Authors:** Yue Wu, Jinwei Pang, Jianhua Peng, Fang Cao, Zongduo Guo, Li Jiang, Zhipeng Teng, Zhijian Huang, Chongjie Cheng, Yong Jiang, Xiaochuan Sun

**Affiliations:** ^1^Department of Neurosurgery, The First Affiliated Hospital of Chongqing Medical University, Chongqing, China; ^2^Department of Neurosurgery, The Affiliated Hospital of Southwest Medical University, Luzhou, China; ^3^Department of Cerebrovascular Disease, The Affiliated Hospital of Zunyi Medical College, Zunyi, China; ^4^Department of Neurosurgery, Chongqing Traditional Chinese Medicine Hospital, Chongqing, China

## Abstract

Neuronal injury is the primary cause of poor outcome after subarachnoid hemorrhage (SAH). The apolipoprotein E (APOE) gene has been suggested to be involved in the prognosis of SAH patients. However, the role of APOE in neuronal injury after SAH has not been well studied. In this study, SAH was induced in APOE-knockout (APOE^−/−^) and wild-type (WT) mice to investigate the impact of APOE deficiency on neuronal injury in the early phase of SAH. The experiments of this study were performed in murine SAH models in vivo and primary cultured microglia and neurons in vitro. The SAH model was induced by endovascular perforation in APOE^−/−^ and APOE WT mice. The mortality rate, weight loss, and neurological deficits were recorded within 72 h after SAH. The neuronal injury was assessed by detecting the neuronal apoptosis and axonal injury. The activation of microglia was assessed by immunofluorescent staining of Iba-1, and clodronate liposomes were used for inhibiting microglial activation. The expression of JNK/c-Jun was evaluated by immunofluorescent staining or western blotting. The expression of TNF-*α*, IL-1*β*, and IL-6 was evaluated by ELISA. Primary cultured microglia were treated with hemoglobin (Hb) in vitro for simulating the pathological process of SAH. SP600125, a JNK inhibitor, was used for evaluating the role of JNK in neuroinflammation. Nitrite production was detected for microglial activation, and flow cytometry was performed to detect apoptosis in vitro. The results suggested that SAH induced early neuronal injury and neurological deficits in mice. APOE deficiency resulted in more severe neurological deficits after SAH in mice. The neurological deficits were associated with exacerbation of neuronal injury, including neuronal apoptosis and axonal injury. Moreover, APOE deficiency enhanced microglial activation and related inflammatory injury on neurons. Inhibition of microglia attenuated neuronal injury in mice, whereas inhibition of JNK inhibited microglia-mediated inflammatory response in vitro. Taken together, JNK/c-Jun was involved in the enhancement of microglia-mediated inflammatory injury in APOE^−/−^ mice. APOE deficiency aggravates neuronal injury which may account for the poor neurological outcomes of APOE^−/−^ mice. The possible protective role of APOE against EBI via the modulation of inflammatory response indicates its potential treatment for SAH.

## 1. Introduction

Subarachnoid hemorrhage (SAH) is a fatal neurovascular disease with an overall mortality of approximately 50%, and more than 30% of survivors remain severely disabled [1]. Over the past decade, more efforts have been made to elucidate the pathophysiological processes of early brain injury (EBI) which is widely accepted as one of the primary causes of poor outcome of SAH [2]. Acute neuronal injury is the core issue of EBI. Integrity of neurons is the basis of intact neurological functions. SAH induces complicated pathological processes including neuroinflammation, oxidative stress, and blood-brain barrier (BBB) interruption, which ultimately aggravate neuronal injury [2]. Therefore, these processes are potential targets for preservation of neuronal function.

Experimental and clinical studies suggest that inflammatory response is a major cause of brain injury after SAH [3–5]. Microglia are primary mediators of the immunological system in the central nervous system (CNS), which rapidly respond to injury and initiate an inflammatory effect [6, 7]. SAH induces activation of resident microglia [8], thereby exerting inflammatory responses in the brain. The roles of microglia in neurological diseases are complicated [9, 10], and whether this cell group is beneficial or harmful is still unclear in the early phase of SAH. Therefore, revealing the role of microglia following SAH provides evidence for targeting immunological cells and their consequent neuroinflammation as a potential treatment for neuronal injury after SAH.

Apolipoprotein E (APOE for gene, ApoE for protein), the major apolipoprotein in the CNS, is a multifunctional protein that is predominantly involved in the transportation of cholesterol and lipid. APOE has been suggested to influence the pathological process of SAH. We have previously found that APOE exerts neuroprotective effects via BBB preservation after SAH [11]. An exogenous ApoE peptide has been demonstrated to improve the neurological functions of SAH mice [12]. Furthermore, in a model of microglial activation, exogenous ApoE inhibited microglial activation and the release of proinflammatory chemicals [13]. Nevertheless, the role of APOE in the process of EBI has not yet been explored. Furthermore, the modulation of SAH-induced inflammatory responses by APOE remains unclear.

Based on the previous reports, we hypothesized that APOE protects against neuronal injury after SAH in an anti-inflammatory manner. To investigate the hypothesis, the impacts of APOE on the neurological outcomes, neuronal damage, and inflammatory responses were assessed in a mouse model of SAH.

## 2. Materials and Methods

### 2.1. Animals

**Figure 1:**
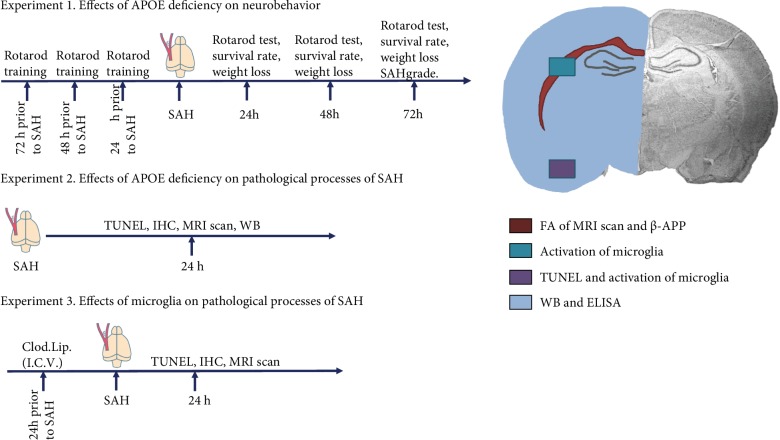
The schema of in vivo study design. SAH: subarachnoid hemorrhage; TUNEL: terminal-deoxynucleoitidyl transferase-mediated nick end labeling; IHC: immunohistochemistry; WB: western blot; Clod. Lip.: clodronate liposome; I.C.V.: intracerebroventricularly.

All experiments were conducted in strict accordance with the recommendations of the Guide for the Care and Use of Laboratory Animals of China. The protocol was approved by the committee on the Ethics of Animal Experiments of Chongqing Medical University. Adult (8-12 weeks) male wild-type (WT) C57BL/6J mice and APOE-knockout (APOE^−/−^) mice on a C57BL/6J background were obtained from the Laboratory Animal Center of Chongqing Medical University. The design of the in vivo study is revealed in Figure 1.

### 2.2. Induction of SAH

SAH induction was performed as previously described [14]. Briefly, animals were anesthetized with an intraperitoneal injection of pentobarbital sodium (50 mg/kg). The right common carotid artery (CCA), external carotid artery (ECA), and internal carotid artery (ICA) were exposed. A 5-0 Prolene filament (Ethicon, Somerville, USA) was advanced into the anterior cerebral artery (ACA) via the ECA and ICA. After a subtle resistance was encountered, the filament was advanced 2 mm further to perforate the ACA. Subsequently, the filament was immediately withdrawn. In the sham operation group, the same procedure was performed with the exception of the perforation of the ACA. The body temperature was maintained at 37.5 ± 0.5°C during the operation. The SAH score is assessed according to a previously reported grading system [15]. Since SAH induces a high mortality rate, mice that died within 72 h were excluded and relevant mice were supplemented.

### 2.3. Rotarod Test and Weight Loss

The rotarod test (TME, Chengdu, China) was used to evaluate the motor deficits of the SAH mice according to the method reported by Hamm et al. [16]. Briefly, all mice were trained at a speed of 16 rpm three times a day for three days prior to SAH induction. Before SAH induction, the baseline of rotarod latency of each mouse was examined with an accelerating speed (starting from 0 rpm, accelerated by 3 rpm every 10 seconds until the rotating speed reached 30 rpm). The accelerating test was repeated at 24 h after SAH induction. Every test was repeated three times. The test ended when mice fell from the rod, and the latencies were recorded. The weight of each mouse was recorded before the rotarod test, and the weight loss was calculated according to the original weight of animals.

### 2.4. Clodronate Liposome Administration

Clodronate liposomes (FormuMax Scientific, Inc., Palo Alto, CA, USA) were injected intracerebroventricularly 1 day prior to SAH induction. Briefly, after mice were anesthetized, a burr hole was drilled 0.22 mm posterior to the bregma, 1 mm lateral, and 2.25 mm in depth to enter the bilateral ventricle. PBS liposomes which do not contain clodronate were used as controls. The dose of clodronate liposomes was adjusted according to the instructions (0.2 ml/20 g).

### 2.5. Cell Culture and Treatment Protocols

Primary microglia were prepared from postnatal day 1 C57BL/6J mice as previously reported [13]. Primary neurons were prepared from a day 15 embryonic cortex obtained from the pregnant C57BL/6J mice as previously reported [17]. All experiments were carried out 24 h after cells were seeded. The cells were treated with hemoglobin (Hb) (Sigma, St. Louis, MO, USA) diluted in culture medium or combined with SP600125 (Abcam, Cambridge, USA) diluted in DMSO for 24 h. The vehicle was used as the control. The supernatants were removed and replaced with fresh DMEM for another 24 h. Then, microglia were collected for assays. The conditioned medium of microglia was collected and added to neurons which were cultured in DMEM/F12 supplemented with 10% FBS for 24 h. Then, the cells were harvested for experiments.

### 2.6. Nitrite Quantification

The production of NO was assessed as the accumulation of nitrite from the spontaneous oxidation of NO in conditioned media after 24 h. Accumulation of nitrite was quantified using a colorimetric reaction with the Griess reagent (Invitrogen, Waltham, MA, USA). Absorbance was measured at 570 nm by spectrophotometry.

### 2.7. Enzyme-Linked Immunosorbent Assay

Quantification of the protein levels of TNF-*α*, IL-1*β*, and IL-6 was performed by the enzyme-linked immunosorbent assay (ELISA). Homogenates of the brain of mice or cultured medium of microglia were prepared for detection according to the manufacturer's instructions of the ELISA kits (Boster, Wuhan, China). The protein content of each sample was detected with a BCA kit (Beyotime, Haimen, China). The results were normalized to protein levels.

### 2.8. Immunofluorescence Staining

Brain samples were fixed with 4% paraformaldehyde for 4 h, followed by overnight immersion in phosphate buffer containing 30% sucrose. The brain samples then were embedded in OCT solution, and coronal frozen sections (10 *μ*m) were prepared. The sections were incubated with primary antibodies at 4°C overnight, including anti-*β*-APP (1 : 100, Abcam, Cambridge, USA), anti-ApoE (1 : 100, Abcam, Cambridge, USA), anti-NeuN (Abcam, Cambridge, USA), anti-Iba-1 (1 : 200, Wako, Osaka, Japan), and anti-P-JNK (Abcam, Cambridge, USA). Sections were then incubated with DyLight 488-conjugated goat anti-rabbit and DyLight 549-conjugated goat anti-mouse secondary antibodies (Abbkine, Redlands, USA). DAPI was used for nuclear staining. The number of positive cells was counted with Image-Pro Plus 6.0 software (Media Cybernetics, Bethesda, USA).

### 2.9. Apoptosis Assay

Coronal frozen sections of around the layer of ICA bifurcation were prepared. A NeuN primary antibody (1 : 100, Abcam, Cambridge, USA) was incubated overnight using the sections prior to TUNEL. TUNEL staining was performed as per the instructions of an in situ cell death detection kit (Roche, Indianapolis, USA). DAPI was used for nuclear staining. TUNEL-NeuN-costained cells were identified as apoptotic neurons with a fluorescence microscope (Leica, Wetzlar, Germany).

### 2.10. MRI Scan

Magnetic resonance imaging (MRI) was performed on a 7.0 T animal scanner (Bruker Biospin, Germany). Mice were anesthetized with 1.5% isoflurane in a mixture of 30% O_2_ and 70% N_2_O and fixed on a holder. Diffusion tensor imaging (DTI) images were acquired using RARE (repetition time = 3000 ms, echo time = 25 ms, field of view = 2.5 cm, and slice thickness = 0.5 mm). A voxel-weighted fractional anisotropy (FA) measure was calculated for the region of the corpus callosum. All images were calculated with Bruker ParaVision 6.0 software (Bruker Biospin, Germany).

### 2.11. Western Blots

Brain hemispheres of mice were homogenized with RIPA (Beyotime, Haimen, Jiangsu, China) plus protease inhibitor cocktail (Roche, Indianapolis, IN, USA) and phosphatase inhibitors (Boster, Wuhan, Hubei, China). Prepared protein extracts were subjected to sodium dodecyl sulfate-polyacrylamide gel electrophoresis and transferred to polyvinylidene difluoride membranes. The membranes were probed overnight at 4°C with the following primary antibodies: anti-ApoE (1 : 500, Abcam, Cambridge, USA), anti-Iba-1 (1 : 500, Wako, Osaka, Japan), anti-P-JNK (1 : 1000, CST, Danvers, MA, USA), anti-JNK (1 : 500, Santa Cruz, Dallas, TX, USA), anti-P-c-Jun (1 : 1000, CST, Danvers, MA, USA), and anti-c-Jun (1 : 1000, CST, Danvers, MA, USA) followed by incubation with secondary antibodies conjugated with horseradish peroxidase. The bands were revealed using an ECL western blotting kit (Thermo Scientific, Pittsburgh, PA, USA) and photographed with a chemiluminescence imaging system (Bio-Rad, Hercules, CA, USA). The amount of protein in each band was quantified using Image Lab Software (Bio-Rad, Hercules, CA, USA).

### 2.12. Flow Cytometry

For apoptotic detection, cultured neurons were harvested after treatment. Neurons were prepared according to the introduction of an apoptosis detection kit (BD, San Jose, CA, USA). Briefly, cells were incubated with 5 *μ*l of Annexin V-FITC dye solution for 15 min at 4°C and 10 *μ*l of PI dye solution for 5 min at room temperature. Then, the cells were subjected to flow cytometry. Annexin V-FITC^+^PI^+^ and Annexin V-FITC^+^PI^−^ cells were deemed apoptotic cells.

### 2.13. Statistical Analysis

Parametric values were expressed as the mean ± standard deviation (SD). Two-tailed Student's *t*-test was used for comparison between two groups, and one-way analysis of variance (ANOVA) was applied for multiple comparisons. Bonferroni's *post hoc* method was applied for comparison among groups. The Fisher exact test was used in two-group comparisons for mortality analysis. All statistic values were calculated using SPSS 19.0 (SPSS, Inc., Chicago, USA). Significance was assumed at *P* < 0.05.

## 3. Results

### 3.1. APOE Deficiency Aggravated Neurological Deficits in the Early Phase of SAH

**Figure 2:**
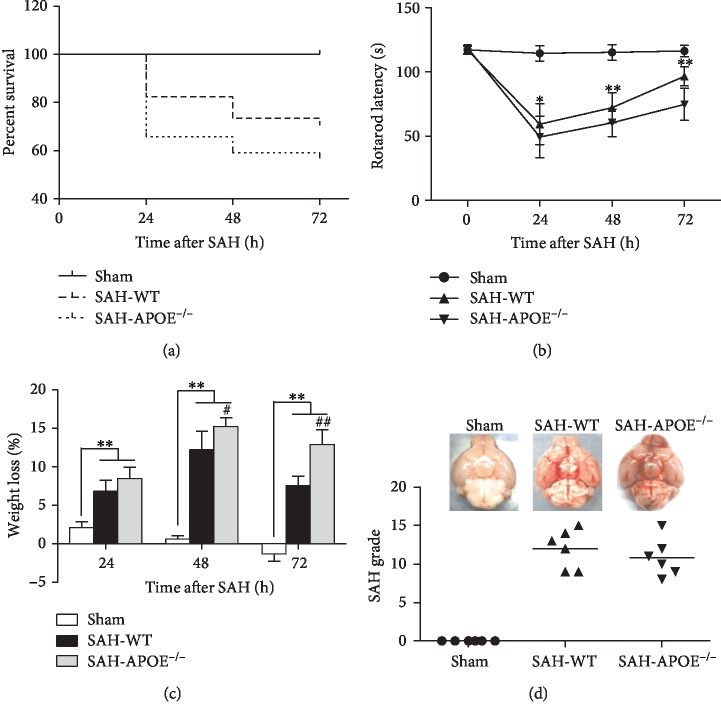
APOE deficiency aggravated neurological deficit within 72 h after SAH. (a) APOE^−/−^ SAH mice exhibited a lower tendency of survival percentage than WT mice. However, the difference was nonsignificant. (b) APOE^−/−^ mice exhibited a more severe motor deficit than WT mice at both time points (^∗^*P* < 0.05, ^∗∗^*P* < 0.01; *n* = 6 for each group). (c) SAH induced weight loss of all mice (^∗∗^*P* < 0.01, compared to sham). Weight loss of APOE^−/−^ mice exceeded that of WT mice at 48 h and 72 h after SAH (^#^*P* < 0.05, ^##^*P* < 0.01; *n* = 6 for each group). (d) No difference was observed between APOE^−/−^ and WT mice in the SAH grade.

In order to investigate the impact of APOE deficiency on early neurological dysfunction after SAH, the mortality rates, rotarod test, and weight loss were assessed in APOE^−/−^ and WT mice. No animal died in the sham-operated group. The overall mortality rate of the WT group within 72 h after SAH was 29.4% (10 of 34), whereas 43.2% of the APOE^−/−^ mice (19 of 44) died within 72 h after SAH (Figure 2(a)). However, the mortality exhibited no significant difference between WT and APOE^−/−^ mice after SAH.

The rotarod latencies of both the APOE^−/−^ and WT mice decreased drastically 24 h after SAH relative to the sham-operated mice, and the neurological functions recovered gradually at 48 h and 72 h after SAH. Meanwhile, APOE^−/−^ mice exhibited worse motor function as indicated by shorter rotarod latencies, relative to WT mice at 24 h, 48 h, and 72 h after SAH (Figure 2(b)). SAH induced weight loss of all mice. Weight loss of APOE^−/−^ mice exceeded that of WT mice at 48 h and 72 h after SAH (Figure 2(c)). No difference was observed between APOE^−/−^ and WT mice in the SAH grade score (Figure 2(d)).

These results revealed that APOE deficiency aggravates neurological deficits in the early phase of SAH. Hence, lacking APOE may cause more severe neuronal damage. To investigate the hypothesis, we further test the neuronal damage in APOE^−/−^ and WT mice after SAH.

### 3.2. APOE Deficiency Aggravated Neuronal Apoptosis and White Matter Injury in the Early Phase of SAH

**Figure 3:**
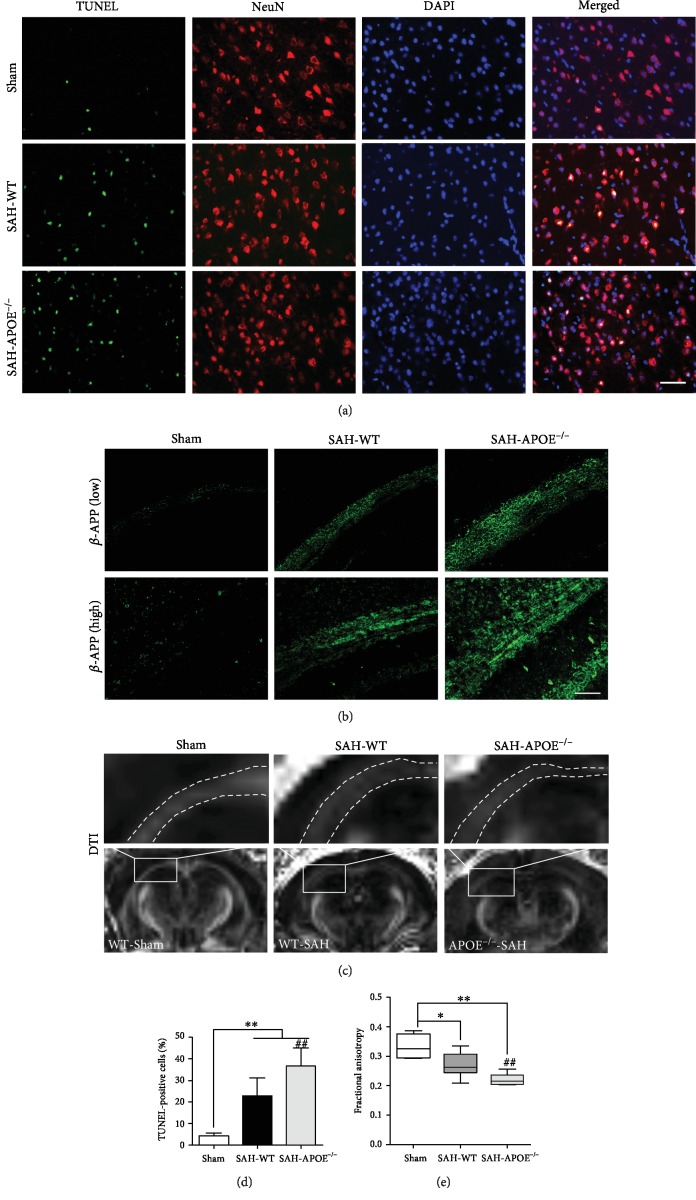
APOE deficiency aggravated neuronal damage in the early phase of SAH. (a) Costaining of TUNEL and NeuN showing apoptotic neurons. (d) SAH induced neuronal apoptosis in the cortex (^∗∗^*P* < 0.01, compared to sham, *n* = 5), while the number of apoptotic neurons of APOE^−/−^ mice was more than that of WT mice (^##^*P* < 0.01). (b) APOE deficiency aggravated *β*-APP accumulation in the white matter after SAH (*n* = 5). Low magnification (200x), high magnification (400x). (c) DTI showing FA decrease after SAH. (e) SAH induced FA decrease (^∗^*P* < 0.05, ^∗∗^*P* < 0.01, compared to sham, *n* = 4), while the FA of APOE^−/−^ mice in the white matter was lower than that of WT mice (^##^*P* < 0.01). Bar = 50 *μ*M.

To reveal the mechanism underlying the varying degrees of neurological deficits between APOE^−/−^ and WT mice, the neuronal damage was investigated. As the neuronal function relies on the integrity of neuronal cell bodies and axons, we further tested neuronal apoptosis and white matter injury in APOE^−/−^ and WT mice at 24 h after SAH. SAH induced evident neuronal apoptosis (Figure 3(a)), while the apoptotic neurons of APOE^−/−^ mice outnumbered those of WT mice (Figure 3(d)). Accumulation of *β*-APP is usually deemed a mark of axonal injury [18]. SAH induced axonal injury in the early phase of SAH, while APOE^−/−^ mice exhibited more severe damage than WT mice after insult (Figure 3(b)). These results provide pathological evidence that APOE^−/−^ mice are more vulnerable in SAH-induced axonal injury. To confirm the pathological findings, the FA in the injured white matter regions was determined with 7.0 T MRI at 24 h after SAH. SAH caused FA decrease in white matter regions (Figure 3(c)), while APOE^−/−^ mice exhibited a greater degree of FA decrease than WT mice (Figure 3(e)).

These results suggested that APOE deficiency exacerbates neuronal damage, including injuries of neuronal cell bodies and axons, which may explain the worse neurological function of APOE^−/−^ mice in the early phase of SAH. We further investigated the mechanisms behind the different degrees of neuronal injury between APOE^−/−^ and WT mice. Based on a previous report, APOE is associated with mediation of microglial activation [13]. Thus, the number of microglia was detected after SAH.

### 3.3. APOE Deficiency Aggravated Microglial Activation

**Figure 4:**
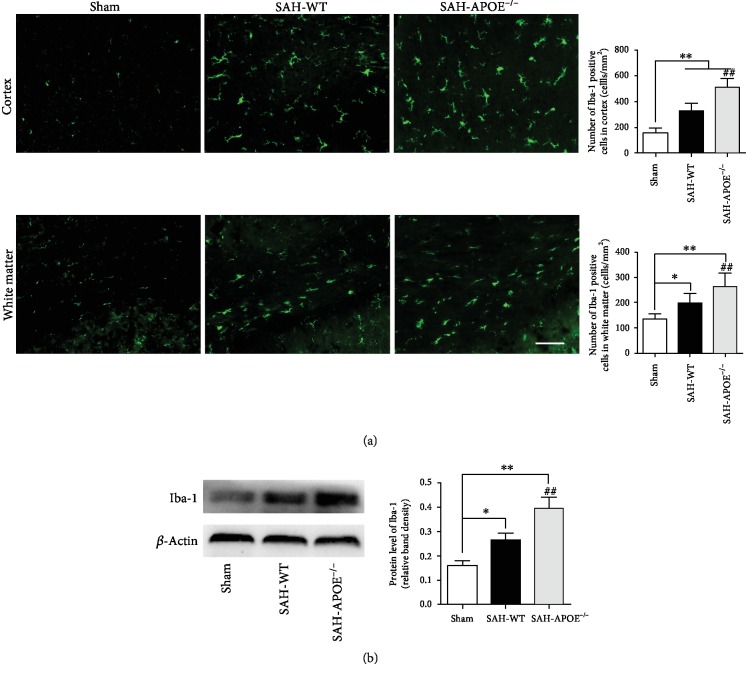
APOE deficiency aggravated microglial activation. (a) SAH increased the number of microglia in the cortex and white matter (^∗^*P* < 0.05, ^∗∗^*P* < 0.01, compared to sham, *n* = 5), while more microglia increased in APOE^−/−^ mice than in WT mice (^##^*P* < 0.01). (b) Western blotting showing overexpression of Iba-1 after SAH (^∗^*P* < 0.05, ^∗∗^*P* < 0.01, compared to sham, *n* = 5). APOE^−/−^ mice exhibited higher Iba-1 level than WT mice (^##^*P* < 0.01). Bar = 50 *μ*M.

Microglia-mediated neuroinflammation is associated with neuronal damage in neurological diseases. A previous report suggests that ApoE inhibits microglial activation in vitro [13]. Our results showed a significant increase in microglia in both the cortex and white matter after SAH. APOE deficiency promoted microglial activation manifesting as more Iba-1-positive cells detected in cortex and white matter SAH (Figure 4(a)). Western blotting confirmed a more active microglial response after SAH (Figure 4(b)).

These results indicated that APOE deficiency aggravated microglial activation, which may exacerbate inflammatory damage of neurons. To verify whether microglial activation is associated with neuronal injury, we further applied clodronate liposome to deplete microglia and detected the neuronal damage after SAH.

### 3.4. Microglial Depletion Alleviated Neuronal Damage

**Figure 5:**
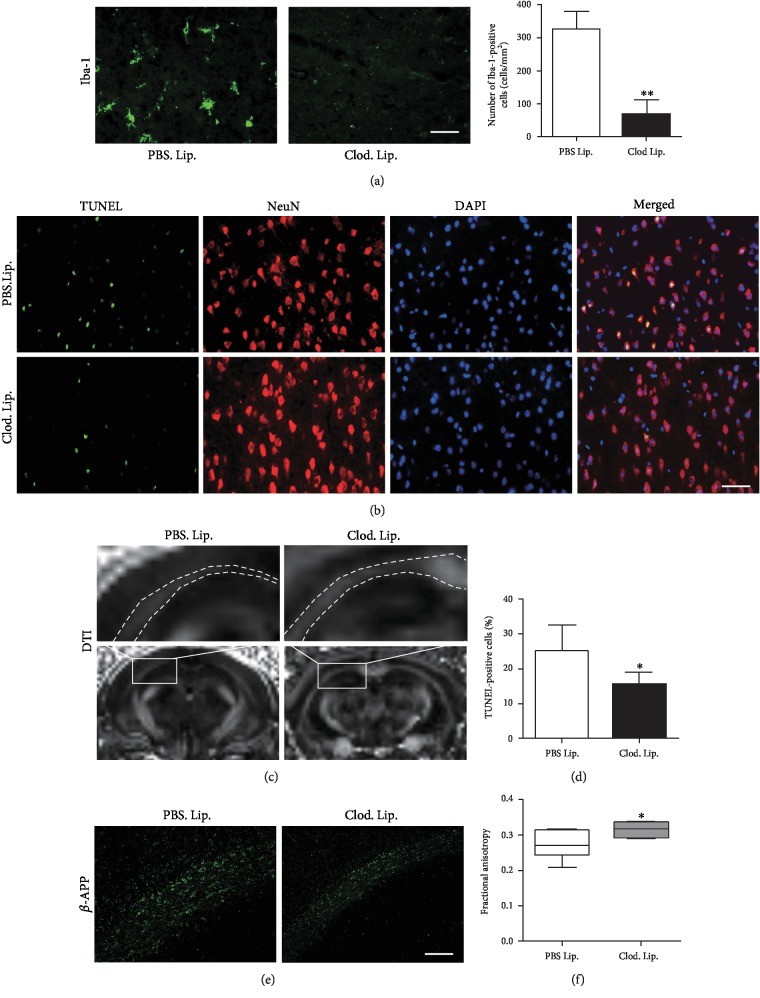
Microglial depletion alleviated neuronal damage. (a) Clod. Lip. reduced Iba-1-positive cells after SAH (^∗∗^*P* < 0.01, *n* = 5). (b, d) Clod. Lip. reduced apoptotic neurons in the cortex after SAH (^∗^*P* < 0.05, *n* = 5). (e) Clod. Lip. inhibited *β*-APP accumulation in the white matter after SAH. (c, f) DTI showing that Clod. Lip. reserved FA in the white matter after SAH (^∗^*P* < 0.05, *n* = 4). Bar = 50 *μ*M.

Clodronate liposome is widely used to deplete microglia and macrophages [9, 19]. A SAH model was applied in WT mice to test the influence of clodronate liposome on neuronal damage. Systemic administration of clodronate liposome reduced the number of microglia in the brain (Figure 5(a)). Clodronate liposome inhibited neuronal apoptosis after SAH (Figures 5(b) and 5(d)). Additionally, clodronate liposome ameliorated *β*-APP accumulation in the white matter after SAH (Figure 5(e)). A DTI image exhibited that clodronate liposome reserved FA (Figures 5(c) and 5(f)), indicating the protection for the white matter by clodronate liposome.

These results showed that microglial depletion alleviates neuronal injury after SAH, which indicates that microglia may mediate inflammatory injury of neurons. To investigate the influence of APOE on microglial inflammatory response, we further detected the expression of inflammation-relative molecules, JNK/c-Jun, and proinflammatory cytokines.

### 3.5. APOE Deficiency Promoted JNK-Mediated Neuroinflammation

**Figure 6:**
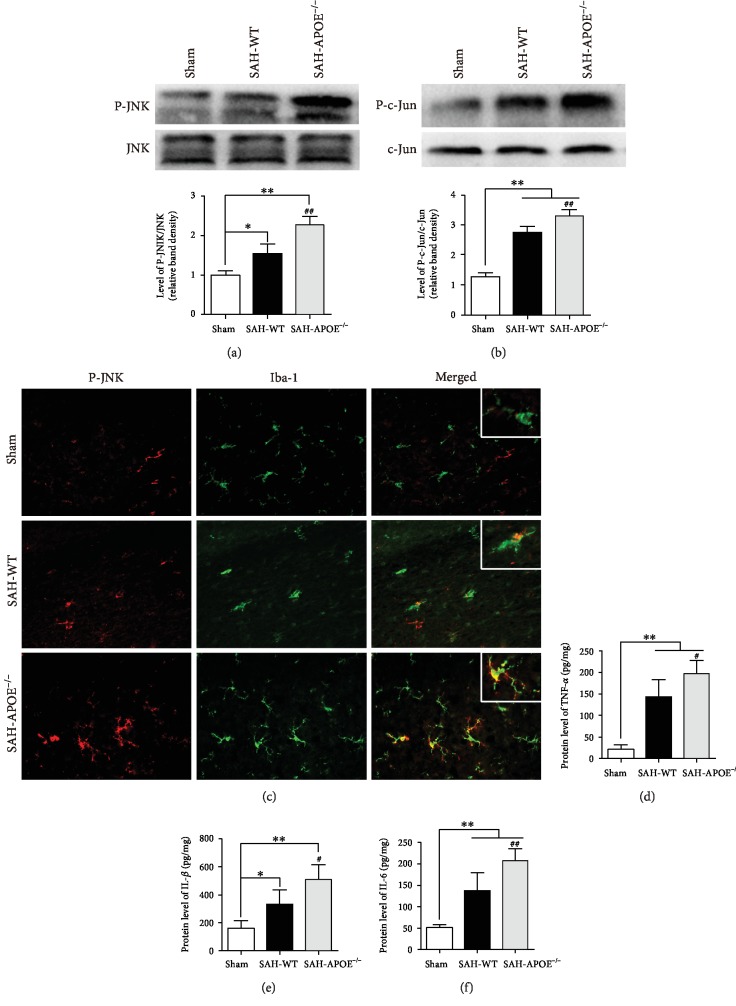
APOE deficiency enhanced JNK-mediated neuroinflammation. (a) SAH promoted JNK phosphorylation (^∗^*P* < 0.05, ^∗∗^*P* < 0.01, compared to sham, *n* = 5), while P-JNK level was higher in APOE^−/−^ mice than in WT mice (^##^*P* < 0.01). (b) SAH promoted c-JUN phosphorylation (^∗∗^*P* < 0.01, compared to sham, *n* = 5), while P-c-Jun level was higher in APOE^−/−^ mice than in WT mice (^##^*P* < 0.01). (c) Immunofluorescence showing higher level of JNK phosphorylation in microglia in APOE^−/−^ mice than in WT mice. (d–f) ELISA showing that SAH promoted cytokine expression including TNF-*α*, IL-1*β*, and IL-6 (^∗^*P* < 0.05,, compared to sham, *n* = 5), while the levels of these cytokines were higher in APOE^−/−^ mice than in WT mice (^#^*P* < 0.05, ^##^*P* < 0.01). Bar = 50 *μ*M.

The findings above indicate that APOE deficiency may aggravate neuronal injury via overactivation of microglia after SAH. The previous report suggests that APOE inhibits microglial activation in vitro via inhibiting the JNK pathway through binding to LRP1, a receptor of ApoE on microglia [20]. Therefore, we evaluated the JNK/c-Jun pathway in SAH mice. SAH promoted JNK and c-Jun phosphorylation, and the phosphorylation levels of JNK and c-Jun in APOE^−/−^ mice were higher than those in WT mice (Figures 6(a) and 6(b)). Costaining was applied to confirm the expression of JNK/c-Jun in microglia. The microglia in sham mice barely expressed P-JNK. SAH promoted JNK phosphorylation in microglia, while APOE deficiency stimulated JNK phosphorylation in microglia after SAH (Figure 6(c)). The expression of downstream proinflammatory cytokines of JNK/c-Jun, TNF-*α*, IL-1*β*, and IL-6, exhibited that APOE deficiency promoted proinflammatory cytokine expression after SAH (Figures 6(d)–6(f)).

These results showed that APOE deficiency promotes JNK/c-Jun activation in microglia and its expression of proinflammatory cytokines after SAH, indicating that APOE may inhibit microglia-induced inflammatory injury on neurons via JNK/c-Jun. To test this hypothesis, we further investigated the role of JNK/c-Jun in inflammatory neuronal injury in vitro.

### 3.6. Microglia Exerted Neuronal Injury via JNK/c-Jun In Vitro

**Figure 7:**
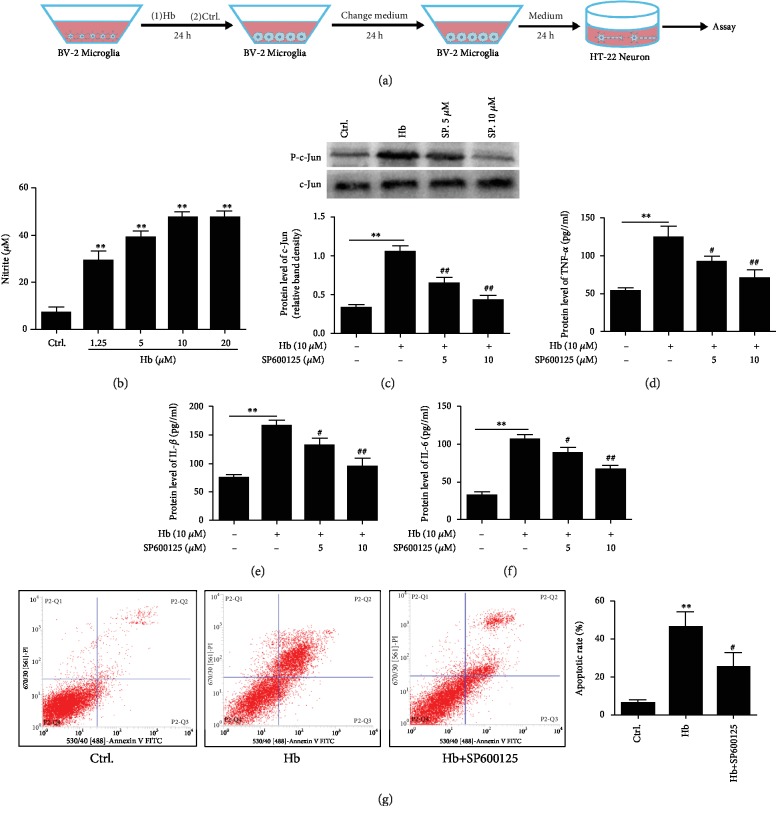
Involvement of JNK in microglial inflammation and neuronal injury. (a) In vitro experiment using cultured microglia incubated with Hb or vehicle control for 24 h. The conditioned medium was applied for incubation of neurons for 24 h. (b) Hb stimulated nitrite expression in microglia. As the doses of Hb increased, the nitrite expression elevated in microglia (^∗∗^*P* < 0.01, *n* = 3). (c) Hb induced phosphorylation of c-Jun in microglia (^∗∗^*P* < 0.01, *n* = 3). SP600125 attenuated c-Jun phosphorylation after Hb incubation (^##^*P* < 0.01, *n* = 3). (d–f) Hb induced cytokine expression including TNF-*α*, IL-1*β*, and IL-6 in microglia (^∗∗^*P* < 0.01, compared to ctrl.), while SP600125 inhibited cytokine expression stimulated by Hb (^#^*P* < 0.05, ^##^*P* < 0.01, *n* = 3). (g) Conditioned medium of Hb-treated microglia induced neuronal apoptosis (^∗∗^*P* < 0.01, *n* = 3), while conditioned medium from SP600125-treated microglia attenuated neuronal apoptosis (^#^*P* < 0.05). Bar = 50 *μ*M.

Hb is one of the major proinflammatory gradients released in the subarachnoid space after SAH [21]. To test the neuronal injury by activated microglia, cultured microglia were treated with Hb or vehicle for 24 h. The supernatants were removed and replaced with fresh DMEM for another 24 h, and then, the cultured medium was collected, respectively, to treat cultured neurons for 24 h (Figure 7(a)). Hb treatment induced microglial activation manifested by the release of the proinflammatory reagent nitrite (Figure 7(b)). Hb treatment stimulated JNK/c-Jun activation, while SP600125, a JNK inhibitor, inhibited the effect of Hb (Figure 7(c)). Additionally, Hb promoted the release of downstream cytokines, TNF-*α* and IL-1*β*, in the medium, while SP600125 reduced cytokine release by microglia (Figures 7(d)–7(f)). These results suggest that microglia-mediated inflammatory response depends on the activation of JNK/c-Jun.

We further investigated the effect of microglia-mediated inflammatory response on neurons by treating them with conditioned medium. Conditioned medium from Hb- (10 *μ*M) treated microglia induced neuronal apoptosis. In contrast, conditioned medium from microglia treated with Hb (10 *μ*M) and SP600125 (10 *μ*M) combination ameliorated neuronal apoptosis (Figure 7(g)). These results showed that JNK/c-Jun was involved in microglia-mediated inflammation which subsequently exerted neuronal injury.

### 3.7. Potential Implication of APOE in Brain Injury in the Early Phase of SAH

**Figure 8:**
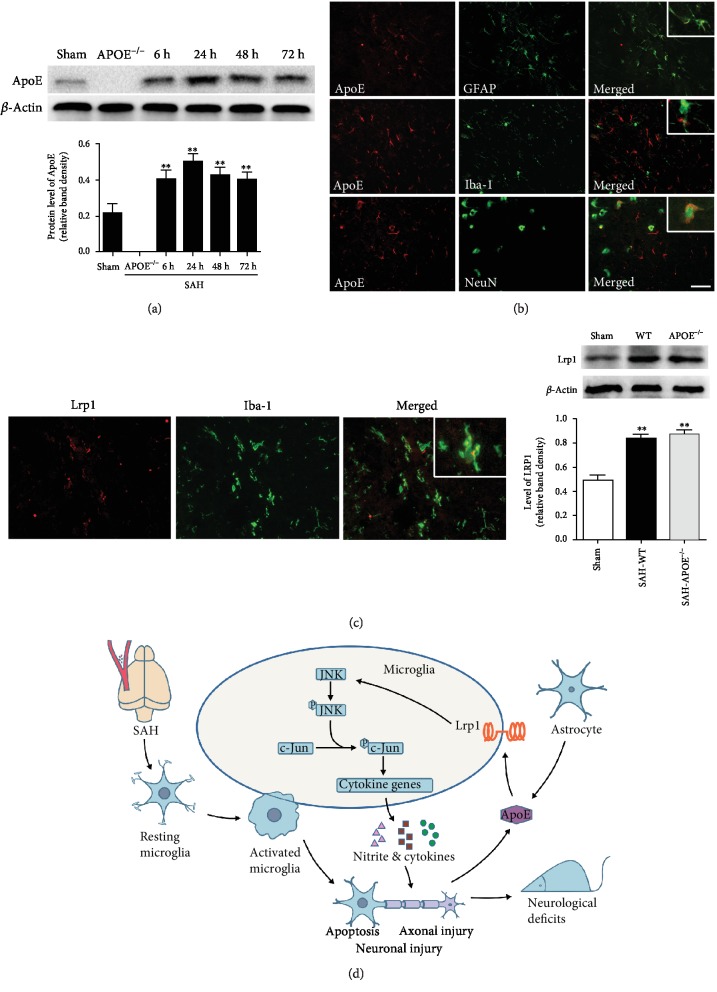
Potential implication of APOE in brain injury in the early phase of SAH. (a) Time course of ApoE expression in the early phase of SAH. The ApoE level elevated after SAH and peaked at 24 h after SAH (^∗∗^*P* < 0.01, compared to sham, *n* = 5). (b) Cellular location of ApoE after SAH. ApoE expressed mainly in astrocytes and partly in neurons. (c) Microglia expressed LRP1. The expression of LRP1 elevated in both APOE^−/−^ and WT mice at 24 h after SAH. (d) Potential mechanisms for involvement of ApoE in brain injury in the early phase of SAH. Bar = 50 *μ*M.

To examine the expression features of ApoE protein after SAH, we further tested the time course of ApoE expression within 72 h after SAH. The results showed an elevation of ApoE expression and peaked at 24 h after SAH, while APOE^−/−^ mice did not express ApoE protein (Figure 8(a)). Costaining of cell markers and ApoE exhibited that ApoE was expressed dominantly in astrocytes and partly in neurons after SAH (Figure 8(b)). Although microglia were not the major source of endogenous ApoE, abundant LRP1, a major receptor of ApoE mediating a JNK/c-Jun signal, was observed in microglia (Figure 8(c)).

Combined with the findings above, APOE deficiency may enhance the JNK/c-Jun activation after SAH, which promotes the microglial inflammatory response. The enhanced neuroinflammation then aggravates neuronal injury and subsequently deteriorates the neurological deficits of APOE^−/−^ mice. In contrast, APOE may exert protection in WT mice after SAH (Figure 8(d)).

## 4. Discussion

In summary, SAH induced neuronal injury and neurological deficits in mice in the early phase. APOE deficiency resulted in more severe neurological deficits after SAH in mice. These deficits were associated with exacerbation of neuronal injury, including neuronal apoptosis and axonal injury. Moreover, APOE deficiency enhanced microglial activation and related inflammatory injury. JNK/c-Jun was involved in the enhancement of inflammatory injury in APOE^−/−^ mice. These results indicate that APOE may exert a protective role against neuronal injury via the suppression of the inflammatory response.

The impact of APOE on the neurological outcomes of SAH animals has been observed in a previous report [22]. The evidence suggests that APOE is involved in the brain injury after SAH. For better understanding of this question, the role of APOE (e.g., protective or damaging) in SAH needs proving. Our results showed that APOE deficiency depleted the endogenous ApoE and its subsequent signaling modulation, which eventually resulted in the aggravation of brain injury. Reversely, we previously found that exogenous ApoE exerts protective effects in SAH mice [12]. These findings suggest that the downstream signaling effect of APOE is protective after SAH. Moreover, it is reported that the different impacts of APOE subtypes on the outcomes of SAH may be due to their diverse affinity to the functional receptors [23, 24]. From this perspective, we speculate that the influence of APOE polymorphisms on the outcomes of SAH may depend on the degrees of neuroprotective effects of different APOE alleles. Combined with the present work, APOE mediates beneficial effects in the early phase of SAH.

SAH has long been discovered to induce neuroinflammatory response [2]. We observed that APOE deficiency increased the microglial count in the brain early after SAH. Microglia play important roles in immunological surveillance and homeostasis maintenance in CNS. Resting microglia that are stimulated by blood cells and lysate transform into an activated phenotype, thereby exerting immunological responses after SAH [8]. It remains unclear whether the microglia in the early phase of SAH are beneficial or harmful. The role of microglia varies in different phases of neurological diseases, which may be due to the complex activating features of these cells [25]. Microglia are observed to transform into different phenotypes which possess totally different biological nature, referred to as microglial polarization. Modulation of microglial polarization is reported to protect against neuronal injury [26]. However, queries suggest that microglia demonstrate a dynamic phenotype determined by the local environment [27]. A black box model may avoid the controversy and assess the comprehensive effect of microglia directly. Hanafy [9] reported that depleting resident microglia by clodronate liposome in mice attenuates neural apoptosis after SAH. In the present study, neuronal injury including apoptosis and white matter injury was attenuated by microglial depletion, indicating that microglia exert a comprehensive harmful effect on neurons in the early phase of SAH.

Elevated levels of inflammatory cytokines are associated with poor outcome of SAH patients in the early phase of SAH [1], suggesting the damaging role of inflammation in SAH. The present findings were consistent with the clinical observation that neuroinflammation contributes to brain injury after SAH. The suppression of the inflammatory response is considered an important aspect of the neuroprotective effects of APOE [28]. For instance, exogenous ApoE attenuates microglial activation and its subsequent inflammatory response in vitro [13]. Additionally, our previous study demonstrated that ApoE inhibits microglial activation and alleviates neuronal damage in EBI [12]. It was observed that the major cellular source of ApoE in the brain is astrocytes, and neurons partly express ApoE. Although microglia barely express ApoE, they are abundant in ApoE receptors. ApoE binds to the functional receptors expressed on the cellular membrane of microglia [29], which may explain the modulation of microglial function by APOE. Besides inflammatory response, oxidative stress may also contribute to the neuronal injury after SAH. Tu et al. [30] reported that ApoE-derived peptide reduces oxidative stress and improves outcome in an ischemic stroke mouse model, indicating that APOE may also regulate oxidative stress in the acute brain pathological process. Chen et al. [31] reported that oxidative stress is related to ROS/JNK signaling pathway acute stress-induced kidney injury. Our study revealed a regulation of the JNK pathway by APOE, which indicates that APOE may modulate oxidative stress via the JNK signaling pathway. The role of oxidative stress in SAH and the possible influence of APOE on oxidative stress need further exploration.

Previous reports have shown that JNK/c-Jun contributes to EBI after SAH [32]. JNK is activated early after SAH, and the inhibition of JNK attenuates apoptosis and BBB interruption. APOE has been demonstrated to suppress microglial activation by inhibiting the JNK pathway in vitro [13]. In the present study, the activation of JNK/c-Jun in microglia was enhanced by APOE deficiency in vivo, while inhibition of JNK phosphorylation alleviated neuronal injury, thereby indicating that APOE attenuates microglia-mediated inflammation, at least in part, via the suppression of the JNK pathway after SAH. Pocivavsek et al. [20] reported that the modulation of microglial inflammation via JNK/c-Jun and the effect are predominantly mediated by LRP1. Moreover, Zhu et al. [33] revealed that ApoE binding to cell surface receptors and the consequential inhibition of JNK/c-Jun activation are required for IL-6, IL-1*β*, and TNF-*α* secretion in macrophages. Higher levels of cytokine production were observed in APOE^−/−^ mice. These cytokines exert inflammatory brain injury in the early phase of SAH. TNF-*α* has been demonstrated to initiate apoptosis by triggering the caspase cascade [27] and mediate myelin and neuronal damage [34]. Microglia are the major source of IL-1 following SAH [35]. IL-1*β* is reported to participate in EBI via activating JNK and MMP-9 [36]. Combined with this evidence, JNK/c-Jun is a key signal in APOE-mediated microglial inflammation after SAH.

Neuronal injury is the major cause of neurological deficits in SAH mice. Axons in the white matter consist in the integrity of neuronal function. White matter injury has recently been reported in SAH mice [37, 38]. Nevertheless, information on how these changes occur is lacking. Mechanical insult from sudden arterial rupture is thought to play a pivotal role in SAH-induced white matter injury, especially in the region distant from the rupture point [38]. More recently, Egashira et al. [39] reported that MMP-9-induced BBB disruption contributes to white matter injury after SAH. Consistent with the study, we found that endogenous ApoE increased as early as 6 h after SAH, and APOE deficiency induced a greater production of IL-1*β* which was previously shown to activate MMP-9 and cause BBB disruption [36]. In the present study, we observed that microglia exert a damaging effect on neurons after Hb treatment, which suggests that inflammation may be responsible for white matter injury after SAH. Wang et al. [10] reported that microglia mediate axonal damage after experimental TBI, which indicates that microglia play important roles in white matter injury. Moreover, we previously observed that microglial response is associated with white matter injury after SAH [40]. Besides the fact that microglia secreting nitrite and cytokines to induce neuronal injury were observed in the present study, it is suggested that microglia may exert direct injury on axons by physical cell-cell interactions [41]. Therefore, we favor the hypothesis that neuroinflammation also contributes to white matter injury, which may be a novel explanation for the vulnerability of APOE^−/−^ mice in SAH, and indicate the prospect of treatment targeting white matter injury.

There are several limitations to the current study. The influence of APOE on brain injury was only tested in the acute-phase post-SAH. APOE may also be involved in the later course of the condition. Therefore, long-term observations should be applied in the future. The semiquantitative nature of the in vivo measurement of microglia is a further limitation. Future in vivo stereological quantifications should be considered. White matter injury was observed in the present study. The mechanisms by which these insults result in EBI and their influences on neurological functions require further exploration.

In conclusion, APOE deficiency aggravates neurological deficits of SAH mice, which may be due to the exacerbation of neuronal apoptosis and white matter injury in the early phase. The aggravated neuronal damage is associated with enhanced microglial activation, which is mediated by APOE via the JNK/c-Jun signal. These results demonstrate the protective role of the APOE gene against neuronal injury and provide evidence for the exploration of APOE-based treatments for SAH.

## Data Availability

All data used to support the findings of this study are available from the corresponding authors upon request.
